# Impact of Environmental and Seasonal Factors on Spontaneous Pneumomediastinum With and Without Pneumorrhachis

**DOI:** 10.1002/kjm2.70096

**Published:** 2025-08-17

**Authors:** Yu‐Wei Liu, Chieh‐Ni Kao, Chi‐Chang Ho, Shah‐Hwa Chou, Pau‐Chung Chen, Shu‐Hung Huang

**Affiliations:** ^1^ PhD Program in Environmental and Occupational Medicine, College of Medicine Kaohsiung Medical University and National Health Research Institutes Kaohsiung Taiwan; ^2^ Division of Thoracic Surgery, Department of Surgery Kaohsiung Medical University, Hospital, Kaohsiung Medical University Kaohsiung Taiwan; ^3^ Department of Surgery, School of Medicine, College of Medicine Kaohsiung Medical University Kaohsiung Taiwan; ^4^ Institute of Environmental and Occupational Health Sciences National Taiwan University College of Public Health Taipei Taiwan; ^5^ Department of Public Health National Taiwan University College of Public Health Taipei Taiwan; ^6^ Department of Environmental and Occupational Medicine National Taiwan University Hospital and National Taiwan University College of Medicine Taipei Taiwan; ^7^ National Institute of Environmental Health Sciences National Health Research Institutes Miaoli Taiwan; ^8^ Division of Plastic Surgery, Department of Surgery Kaohsiung Municipal Siaogang Hospital Kaohsiung Taiwan; ^9^ Graduate Institute of Medicine, College of Medicine Kaohsiung Medical University Kaohsiung Taiwan

**Keywords:** air pollution, environmental factors, pneumorrhachis, seasonal variations, spontaneous pneumomediastinum

## Abstract

Spontaneous pneumomediastinum (SPM) with pneumorrhachis is rare but generally benign and self‐limiting. However, the impact of environmental and seasonal factors on SPM remains unclear. This study investigated their association with SPM onset and clinical outcomes. We conducted a 12‐year retrospective review of SPM cases, comparing clinical characteristics and outcomes between patients with and without pneumorrhachis. A case‐crossover design was used to assess short‐term associations between environmental exposures and SPM incidence, analyzed via conditional logistic regression. A total of 70 consecutive patients were identified, with 9 classified as SPM with pneumorrhachis and 61 as SPM without pneumorrhachis. While both groups were predominantly managed with hospitalization, those with pneumorrhachis had longer hospital stays (median: 7 vs. 3 days, *p* = 0.002) and were more often associated with severe‐grade SPM and identifiable triggers (*p* < 0.001 and *p* = 0.009, respectively). No significant environmental exposure differences were observed between groups. Seasonally, SPM incidence was significantly higher in autumn and winter (*p* < 0.001), consistent with elevated air pollutant levels. Linear regression showed that standardized *β* coefficients for PM_2.5_ were higher in autumn and winter (*β* = 1.15 and *β* = 1.18), indicating a seasonal association between PM_2.5_ and SPM onset. Despite experiencing more triggers and longer hospitalization, patients with pneumorrhachis had similarly favorable clinical courses. The seasonal clustering of SPM and its association with elevated PM_2.5_ levels suggest that air pollution may be a contributing factor, warranting further investigation.

## 1 Introduction

Spontaneous pneumomediastinum (SPM) is defined as the presence of free air within the mediastinal space occurring without preceding trauma, iatrogenic intervention, or secondary causes such as esophageal perforation [1, 2, 3]. A substantial proportion of SPM cases result from a sudden, rapid increase in intrathoracic pressure. Common triggers include Valsalva maneuvers, strenuous physical exertion, forceful coughing, vomiting, illicit drug consumption, and asthma exacerbations [1, 2, 3, 4, 5, 6, 7, 8, 9, 10, 11]. The underlying mechanism involves alveolar rupture, followed by the tracking of air along the bronchovascular sheaths into the mediastinum, known as the Macklin effect [12]. However, a significant number of patients present without identifiable precipitating factors [2, 4, 7, 8], suggesting that additional, less well‐understood mechanisms may contribute to the pathogenesis of SPM.

Currently, no established guidelines exist for the diagnosis and treatment of spontaneous pneumomediastinum (SPM) [2, 3, 4]. Recent studies suggest that extensive diagnostic workups may be unnecessary, as SPM generally follows a benign clinical course [10, 11]. However, the severity of pneumomediastinum remains uncertain, with a few studies classifying its severity based on the extent of air distribution in the mediastinum and subcutaneous emphysema [2, 13]. Pneumorrhachis, characterized by air in the epidural or intradural spaces of the spinal canal, has been associated with more severe cases of pneumomediastinum [13, 14]. The reported incidence of pneumorrhachis among SPM patients ranges from 5.8% to 9.5% [13, 15]. Yet, its true prevalence may be underestimated due to inconsistent CT scan utilization among clinicians and age‐related differences in imaging practices [13]. In contrast, the triggers for similar respiratory conditions, such as spontaneous pneumothorax (SP), have not been sufficiently evaluated. Over the past few decades, studies have explored potential associations between SP incidence and meteorological or environmental factors [16, 17, 18, 19]. Nevertheless, existing research of SPM primarily focuses on the causes, clinical presentation, and management, with no dedicated analysis of seasonal patterns or environmental impact [1, 2, 3, 4, 5, 6, 7, 8, 9, 10, 11, 12].

Therefore, this study aimed to (1) evaluate the association between environmental factors, seasonal variation, and SPM occurrence in a relatively large cohort at a single center over a 12‐year period and (2) assess clinical outcomes associated with SPM complicated by pneumorrhachis.

## 2 Methods

### 2.1 Study Design and Patient Enrollment

The medical records of patients who were diagnosed with pneumomediastinum and treated at a tertiary center in southern Taiwan were retrospectively analyzed. The diagnosis of pneumomediastinum was based on the finding of free air in the mediastinum on chest X‐ray and chest computed tomography (CT) images. Patients who had traumatic pneumomediastinum or iatrogenic pneumomediastinum following invasive investigations, had spontaneous pneumomediastinum but without a CT scan, or who were transferred to other hospitals were excluded from this study. Therefore, only those who were diagnosed with spontaneous pneumomediastinum (SPM) and were admitted to our hospital for treatment were included in this study.

### 2.2 Severity of Pneumomediastinum Classified by CT Scan and Definition of Pneumorrhachis

**FIGURE 1:**
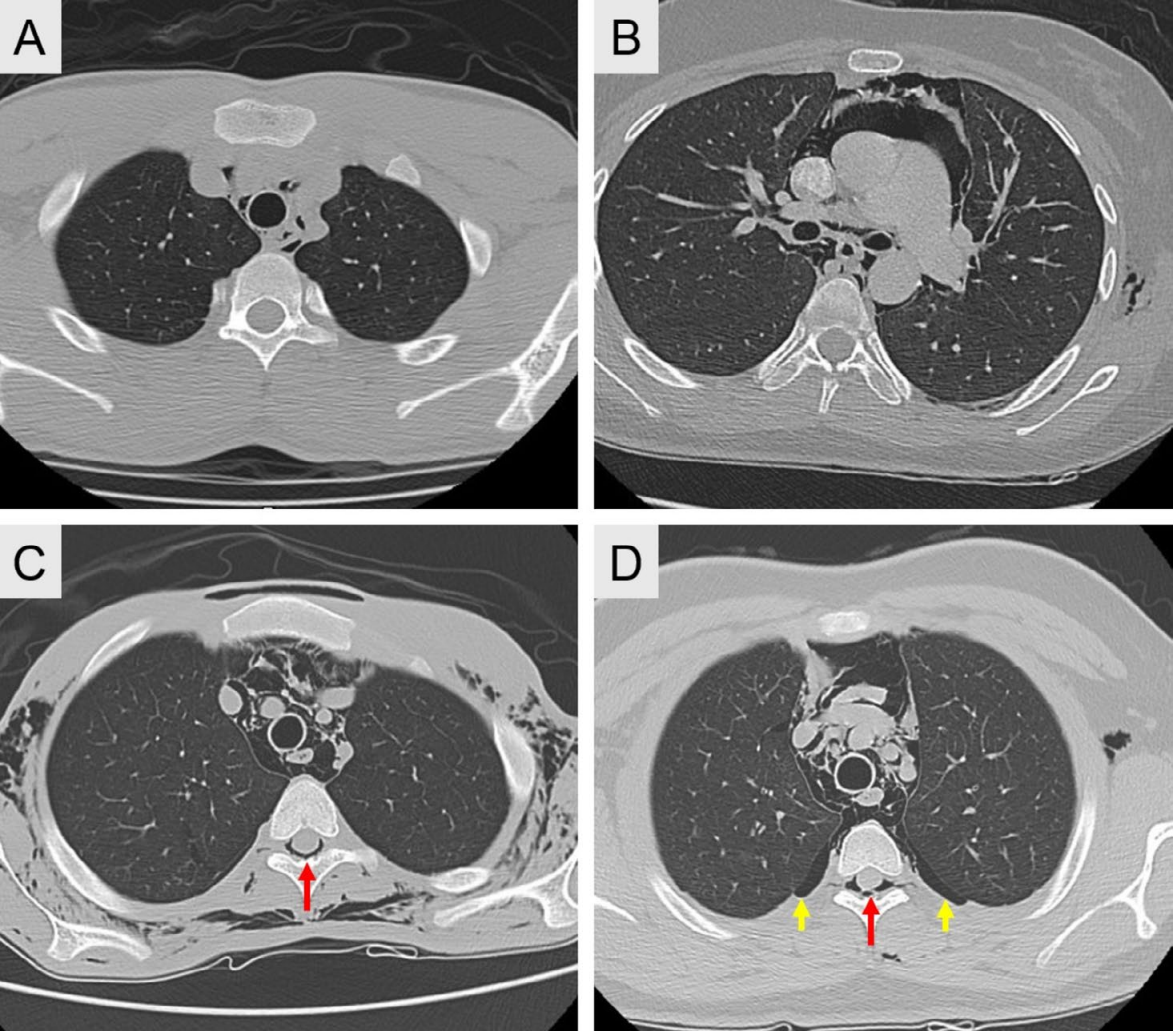
Representative computed tomography (CT) images of spontaneous pneumomediastinum (SPM) cases, classified by severity criteria. (A) Mild (Grade A): Minimal mediastinal air extending from the larynx to the carina, with limited cervical subcutaneous emphysema. (B) Moderate (Grade B): Features of mild SPM plus involvement of the peribronchial tree and pericardial region, along with subcutaneous emphysema. (C) Severe (Grade C): Features of moderate SPM accompanied by extensive chest wall subcutaneous emphysema and coexisting pneumorrhachis (indicated by red arrow). (D) SPM with concurrent subcutaneous emphysema, pneumorrhachis (*red arrow*), and pneumothorax (*yellow arrow*). SPM, spontaneous pneumomediastinum.

According to the classification proposed by Kim et al. [2] in 2015 and by Behr et al. [13] in 2018, each CT scan was reviewed by a subspecialty radiologist to identify the degree of severity of SPM. The severity of SPM was graded according to the distribution of air in the mediastinum and the extent of subcutaneous emphysema accordingly [2, 13]. Grade A (mild) showed the presence of minimal air shadow from the larynx to the carina level plus restricted cervical subcutaneous emphysema; grade B (moderate) showed mild criteria plus involvement of the peribronchial tree and pericardial area with subcutaneous emphysema; and grade C (severe) showed moderate criteria plus extensive chest wall subcutaneous emphysema. Spontaneous pneumorrhachis is a rare condition and occasionally correlates with SPM [13, 15]. It is hypothesized to originate from an air leak spreading through the posterior mediastinum into the epidural space via the cervical fascia planes or neural foramen [20]. The representation of SPM with or without pneumorrhachis is illustrated in Figure 1.

The demographics of the patients, trigger factors, investigation findings, the management, and clinical course were analyzed. The study was conducted in accordance with the Declaration of Helsinki and was approved by the Ethics Committee of Kaohsiung Medical University Hospital (Approval no. KMUHIRB‐E(I)‐20180071), with a waiver of written informed consent.

### 2.3 Exposure Measurement

The environmental data were sourced from the Taiwan Climate Change Projection Information and Adaptation Knowledge Platform (TCCIP) [21], while the air pollution data were provided by the Environmental Protection Administration. Daily air pollutant concentrations were estimated via hybrid spatial prediction models on the basis of measurements from Taiwan Air Quality Monitoring (TAQM) stations. Data on meteorological and air pollution factors were collected for patients' residential areas and regular activity regions, with simulated data estimated to the township level.

The key pollutants analyzed included NO_2_, O_3_, and PM_2.5_, alongside meteorological factors such as atmospheric pressure, temperature, rainfall, and relative humidity. The time intervals for these factors were extracted to explore associations with SPM onset. Detailed methodologies for air pollutant data collection, geospatial datasets, and prediction models have been published previously [22].

A case‐crossover design was used, allowing patients to serve as their own controls. This method compares data from the same individual at different time points, effectively controlling for time‐invariant confounders like genetics, sex, and chronic diseases. By minimizing inter‐individual variability, it reduces confounding effects on study outcomes. While robust against certain biases, this retrospective approach may overlook time‐varying confounders, such as acute disease onset. However, such events are generally rare and unlikely to significantly affect the causal inference between air pollution and health outcomes.

In terms of posttreatment follow‐up, patients attended outpatient clinics 1 week post‐discharge. They were instructed to seek medical attention if they experienced common symptoms suggestive of spontaneous pneumomediastinum (SPM) recurrence, such as chest pain, dyspnea, cough, or vomiting.

### 2.4 Statistical Analysis

**FIGURE 2:**
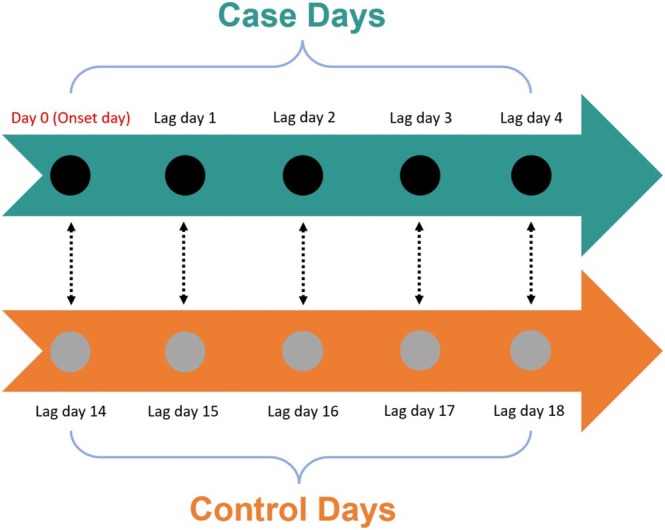
A scheme showing case days and control days related to case–control design. The case day (Day 0) represents the date of SPM symptom onset. Lag days 1–4, representing the four days preceding symptom onset, were also analyzed to assess delayed exposure effects. Control days were unidirectionally matched and selected 14–18 days prior to the case day to evaluate temporal associations and minimize confounding.

Statistical analyses were performed using STATA 14.0 for Windows. In the case‐crossover design, patients served as their own controls. Continuous variables are presented as means ± standard deviations (SDs) or medians (ranges), while categorical variables are shown as percentages. Categorical variables were compared using the chi‐square test or Fisher's exact test. Spearman's rank correlation coefficient assessed relationships between meteorological factors and air pollutants. The case day was defined as the day of SPM symptom onset (day 0). Lag days 1–4 (preceding symptom onset) were also analyzed, with unidirectional matched control days selected 14–18 days prior to the case day to evaluate lag effects (Figure 2). Conditional logistic regression was employed for unidirectionally matched data, with odds ratios (ORs) reported per standard deviation increase in daily mean or maximum values. In addition, standardized beta coefficients (*β*) were used to compare the relative influence of air pollutant levels, as all predictors were standardized to a mean of 0 and a standard deviation of 1 before regression analysis. The results are presented with 95% confidence intervals (CIs), and statistical significance was set at *p* < 0.05.

## 3 Results

### 3.1 Clinical Characteristics

**FIGURE 3:**
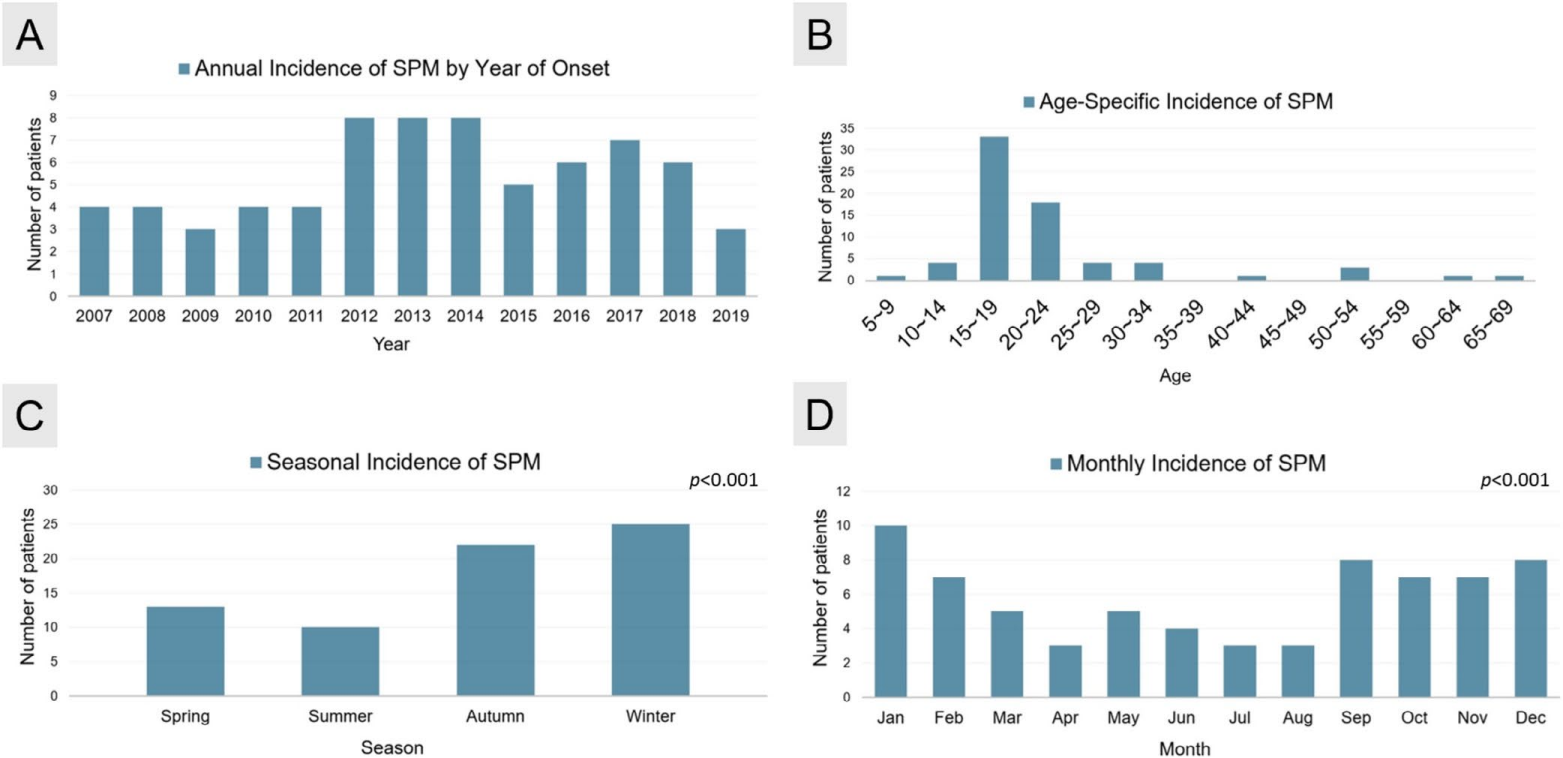
Distribution of SPM by year, age, season, and month. (A) Incidence of SPM by year of onset. (B) Incidence of SPM by specific age group. (C) Incidence of SPM by season. (D) Incidence of SPM by month. SPM, spontaneous pneumomediastinum.

Between January 2007 and January 2019, a total of 139 patients were diagnosed and treated for pneumomediastinum in the thoracic surgery division of our hospital. Among them, patients with trauma‐related pneumomediastinum or iatrogenic pneumomediastinum resulting from invasive procedures (*n* = 45) were excluded. Additionally, those with underlying conditions such as interstitial pneumonia, chronic obstructive pulmonary disease (COPD), or tumors in the aerodigestive tract were not included (*n* = 12). Patients who did not undergo chest computed tomography (CT) scans (*n* = 7) and those transferred to other hospitals (*n* = 5) were also excluded. After applying these exclusion criteria, a total of 70 patients were classified as having spontaneous pneumomediastinum (SPM) and included in the study over a 12‐year timespan (see Figure 3). The median age of patients at the SPM onset was 17 years (range: 5–65 years). Among these, 57 patients (81.4%) were male, and 10 (14.3%) were smokers.

These patients were further categorized into SPM with pneumorrhachis (*n* = 9) and SPM without pneumorrhachis (*n* = 61). As shown in Table 1, a comparison between pneumorrhachis and non‐pneumorrhachis patients revealed no significant differences in age, sex, smoking status, BMI, or clinical investigations and treatments received. However, patients with pneumorrhachis had a longer hospital stay, a higher proportion of severe‐grade SPM cases, and were more likely to have identified triggering events (*p* = 0.002, *p* < 0.001, and *p* = 0.009, respectively). Although recurrence was rare (2/70, 2.8%), no significant difference in recurrence rates was observed between the two groups.

**TABLE 1:** Comparison of SPM patient characteristics based on pneumorrhachis.

Variables	SPM patients (*n* = 70)	SPM without Pneumorrhachis (*n* = 61)	SPM with Pneumorrhachis (*n* = 9)	*p*
Age (year)				
Median (range)	17 (5–65)	17.0 (9–64)	18.0 (5–65)	0.98
Mean (SD)	21.10 (11.4)	20.7 (10.1)	23.9 (18.2)	
Male, No. (%)	57 (81.4%)	49 (80.3%)	8 (88.9%)	0.54
Smoking, No. (%)	10 (14.3%)	8 (13.1%)	2 (22.2%)	0.61
Weight (Kg)				
Median (range)	55.5 (17.5–91.0)	55.0 (26.0–91.0)	56.5 (17.5–90.0)	0.87
Mean (SD)	57.12 (14.1)	57.3 (13.2)	55.6 (19.7)	
Height (cm)				
Median (range)	168 (105–186)	169.0 (126–186)	164.5 (105–180)	0.29
Mean (SD)	165.5 (12.9)	166.4 (10.9)	159.1 (22.3)	
Body mass index (Kg/m^2^)				
Median (range)	20.4 (12.2–32.8)	20.3 (12.2–32.8)	21.8 (15.9–31.5)	0.76
Mean (SD)	20.6 (3.8)	20.5 (3.6)	21.3 (4.8)	
Triggering event (yes) No. (%)	32 (45.7%)	24 (39.3%)	8 (88.8%)	
Asthma attack	8 (11.4%)	5 (8.2%)	3 (33.3%)	0.009
Blowing saxophone	1 (1.4%)	1 (1.6%)	0	
Strenuous exercise	5 (7.1%)	5 (8.2%)	0	
URI	9 (12.9%)	6 (9.8%)	3 (33.3%)	
Vigorous cough	5 (7.1%)	4 (6.6%)	1 (11.1%)	
Vomiting	4 (5.7%)	3 (4.9%)	1 (11.1%)	
Hospital stay (day)				
Median (range)	4 (2–37)	3.0 (2.0–37.0)	7.0 (4.0–28.0)	0.002
Mean (SD)	5.7 (6.2)	5.6 (5.9)	6.9 (8.1)	
Severity, No. (%)				
A	20 (28.6%)	20 (32.8%)	0	< 0.001
B	32 (45.7%)	32 (52.2%)	0	
C	18 (25.7%)	9 (14.8%)	9 (100.0%)	
Clinical investigation and therapy				
Esophagram, No. (%)	44 (62.9%)	39 (63.9%)	5 (55.6%)	0.63
Bronchoscopy, No. (%)	14 (20.0%)	12 (19.2%)	2 (22.2%)	0.86
EGD, No. (%)	5 (7.1%)	5 (8.2%)	0	0.37
Antibiotic therapy, No. (%)	46 (65.7%)	38 (62.3%)	8 (88.9%)	0.15
Oxygen therapy, No. (%)	70 (100.0%)	61 (100.0%)	9 (100.0%)	0.99
Recurrence, No. (%)	2 (2.8%)	2 (3.3%)	0	0.58

Abbreviations: EGD, esophagogastroduodenoscopy; SPM, spontaneous pneumomediastinum.

### 3.2 Environmental Factors Associated With SPM Onset

**FIGURE 4:**
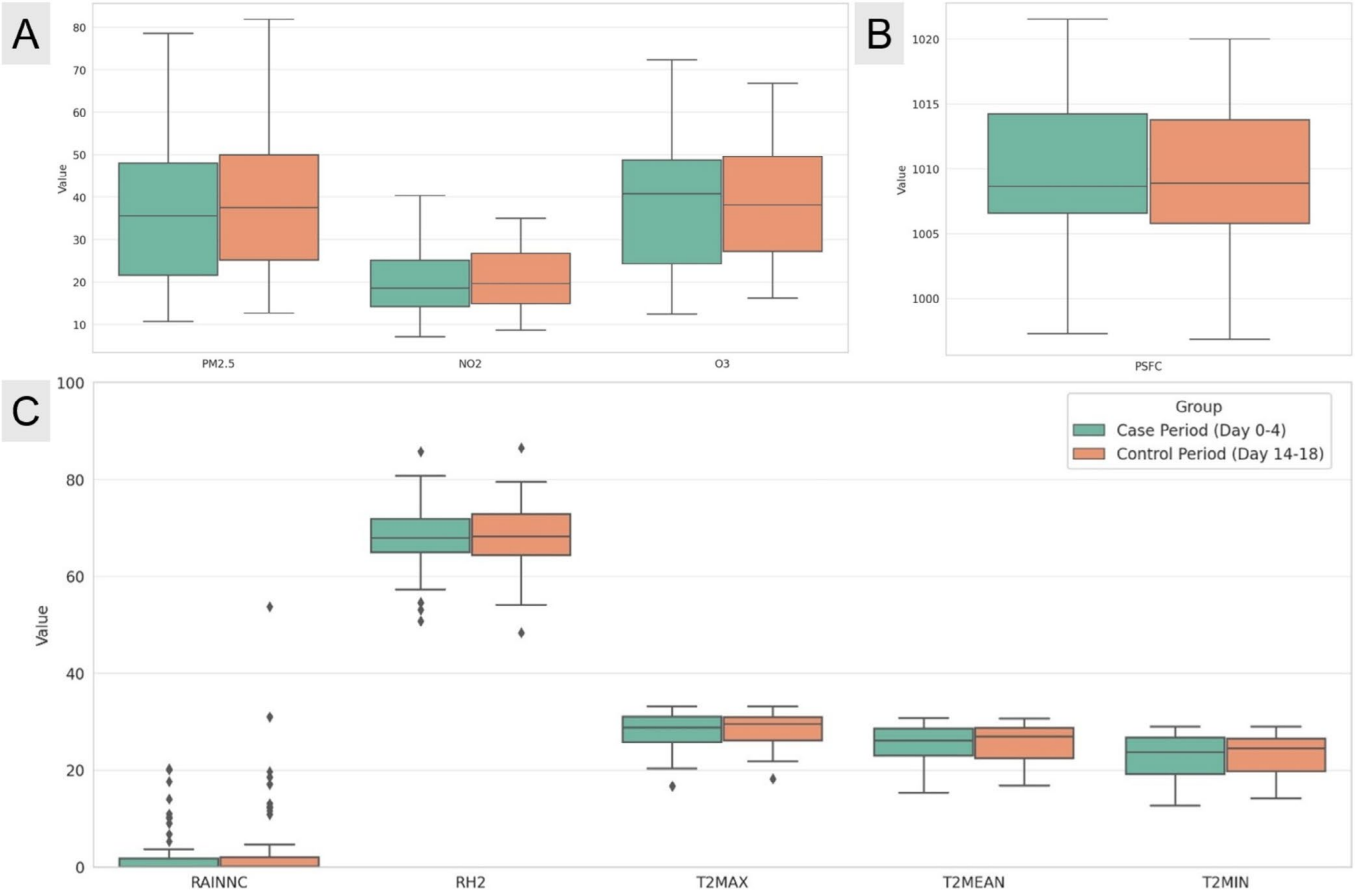
Box plot analysis showing the distributions of daily average air pollutant levels and meteorological factors on days of SPM occurrence. (A) Distribution of air pollutant levels on days when spontaneous pneumomediastinum (SPM) occurred. (B) Distribution of daily mean surface pressure (PSFC) associated with SPM occurrence. (C) Distribution of daily average meteorological parameters on SPM days, including accumulated non‐convective precipitation (RAINNC), relative humidity at 2 m (RH_2_), and temperature variables (maximum T2MAX, mean T2MEAN, and minimum T2MIN) at 2 m above ground. PSFC, surface pressure; RAINNC, accumulated non‐convective precipitation; RH2, relative humidity at 2 m above ground; SPM, spontaneous pneumomediastinum; T2MAX, maximum temperature at 2 m above ground; T2MEAN, mean temperature at 2 m above ground; T2MIN, minimum temperature at 2 m above ground.

The daily mean levels of PM_2.5_, NO_2_, and O_3_ on the day of SPM onset were 37.9 μg/m^3^, 20.4 ppb, and 38.1 ppb, respectively. The meteorological parameters on the day of SPM onset included a daily mean surface atmospheric pressure of 1009.9 hPa, rainfall of 5.5 mm, relative humidity of 68.0%, and temperature of 25.3°C. The distributions of daily average air pollutant levels and meteorological factors for SPM are shown with visual representations provided in Figure 4.

Tables 2 and 3 illustrate the relationships between SPM and both air pollutant levels and meteorological factors. However, neither air pollutant levels nor meteorological factors showed significant associations with SPM onset across all lag days.

**TABLE 2:** Univariate regression analysis of air pollutant levels as predictors of SPM.

Factors	PM_2.5_ (μg/m^3^)		NO_2_ (ppb)		O_3_ (ppb)	
	OR (95% CI)	*p*	OR (95% CI)	*p*	OR (95% CI)	*p*
Case period (0–4 Day)	0.99 (0.97–1.02)	0.61	0.99 (0.94–1.05)	0.85	1.00 (0.98–1.03)	0.80
Lag 0	0.99 (0.97–1.02)	0.58	0.99 (0.95–1.05)	0.86	1.00 (0.98–1.03)	0.70
Lag 1	0.99 (0.98–1.02)	0.99	1.0 (0.95–1.05)	0.98	1.01 (0.99–1.03)	0.49
Lag 2	0.99 (0.97–1.01)	0.49	1.0 (0.95–1.05)	0.97	1.0 (0.98–1.03)	0.84
Lag 3	0.99 (0.98–1.02)	0.84	0.99 (0.94–1.04)	0.70	1.01 (0.98–1.03)	0.65
Lag 4	0.99 (0.97–1.02)	0.59	0.99 (0.94–1.04)	0.69	0.99 (0.97–1.02)	0.60

Abbreviation: SPM, spontaneous pneumomediastinum.

**TABLE 3:** Univariate regression analysis of meteorological factors as predictors of SPM.

Factors	Daily mean surface pressure (hPa)		Daily average rainfall (mm)		Daily average relative humidity (%)		Daily maximum temperature (°C)		Daily average temperature (°C)		Daily minimum temperature (°C)	
	OR (95% CI)	*p*	OR (95% CI)	*p*	OR (95% CI)	*p*	OR (95% CI)	*p*	OR (95% CI)	*p*	OR (95% CI)	*p*
Case period (0–4 Day)	1.02 (0.95–1.09)	0.59	1.01 (0.98–1.03)	0.61	0.99 (0.94–1.05)	0.84	0.98 (0.89–1.08)	0.67	0.97 (0.89–1.07)	0.56	0.98 (0.90–1.05)	0.53
Lag 0	1.02 (0.96–1.09)	0.48	0.98 (0.94–1.02)	0.39	0.99 (0.95–1.05)	0.84	0.98 (0.89–1.09)	0.75	0.98 (0.89–1.08)	0.69	0.99 (0.91–1.07)	0.71
Lag 1	1.03 (0.96–1.09)	0.44	1.01 (0.99–1.03)	0.56	0.99 (0.94–1.04)	0.71	0.99 (0.89–1.09)	0.76	0.98 (0.89–1.07)	0.64	0.97 (0.89–1.05)	0.49
Lag 2	1.03 (0.96–1.10)	0.37	1.00 (0.97–1.03)	0.74	0.99 (0.95–1.04)	0.86	0.96 (0.88–1.06)	0.43	0.96 (0.88–1.05)	0.37	0.97 (0.89–1.04)	0.39
Lag 3	1.01 (0.95–1.08)	0.66	1.00 (0.99–1.02)	0.51	0.99 (0.95–1.04)	0.80	0.98 (0.89–1.08)	0.67	0.97 (0.89–1.06)	0.51	0.97 (0.90–1.05)	0.44
Lag 4	1.02 (0.96–1.09)	0.57	1.01 (0.96–1.03)	0.44	0.99 (0.95–1.05)	0.75	0.97 (0.89–1.08)	0.62	0.97 (0.88–1.06)	0.51	0.98 (0.91–1.04)	0.62

Abbreviation: SPM, spontaneous pneumomediastinum.

Moreover, comparison of daily air pollutant levels and weather data between SPM patients with and without pneumorrhachis showed no significant environmental differences between groups (see Table 4).

**TABLE 4:** Comparison of daily air pollutant levels and weather data between SPM patients with and without pneumorrhachis.

Variables	SPM without pneumorrhachis (*n* = 61)	SPM with pneumorrhachis (*n* = 9)	*p*
Lag 0			
PM_2.5_ (μg/m^3^)			
Median (range)	32.5 (10.8–78.6)	37.8 (14.3–81.4)	0.07
NO_2_ (ppb)			
Median (range)	18.4 (7.1–40.4)	24.5 (9.9–42.7)	0.12
O3 (ppb)			
Median (range)	31.8 (14.1–72.3)	35.1 (12.4–54.7)	0.60
Daily mean surface pressure (hPa)			
Median (range)	1010.04 (995.46–1022.25)	1009.16 (1006.74–1019.35)	0.42
Daily average rainfall (mm)			
Median (range)	0.00 (0.00–9.96)	0.00 (0.00–0.59)	0.55
Daily average relative humidity (%)			
Median (range)	68.81 (44.53–85.80)	66.22 (61.73–71.33)	0.57
Daily maximum temperature (°C)			
Median (range)	28.89 (17.09–34.11)	28.99 (23.03–32.88)	0.85
Daily average temperature (°C)			
Median (range)	26.49 (16.01–30.95)	25.85 (19.37–30.59)	0.86
Daily minimum temperature (°C)			
Median (range)	24.08 (13.52–29.22)	22.69 (16.76–28.26)	0.83
Lag 1			
PM_2.5_ (μg/m^3^)			
Median (range)	36.7 (12.1–78.3)	37.6 (16.9–70.9)	0.21
NO_2_ (ppb)			
Median (range)	19.7 (6.5–35.4)	23.9 (9.5–34.3)	0.16
O_3_ (ppb)			
Median (range)	35.9 (8.5–71.2)	38.1 (29.8–74.8)	0.82
Daily mean surface pressure (hPa)			
Median (range)	1008.60 (997.75–1022.76)	1009.23 (1003.61–1018.64)	0.61
Daily average rainfall (mm)			
Median (range)	0.00 (0.00–175.23)	0.00 (0.00–5.56)	0.55
Daily average relative humidity (%)			
Median (range)	68.69 (47.47–88.19)	67.44 (59.63–69.47)	0.50
Daily maximum temperature (°C)			
Median (range)	29.45 (18.40–33.08)	30.06 (23.91–32.54)	0.60
Daily average temperature (°C)			
Median (range)	26.79 (16.75–30.61)	25.78 (19.85–30.17)	0.98
Daily minimum temperature (°C)			
Median (range)	24.02 (13.99–29.02)	21.77 (16.58–28.00)	0.68
Lag 2			
PM_2.5_ (μg/m^3^)			
Median (range)	35.6 (12.3–67.5)	40.1 (19.4–65.3)	0.24
NO_2_ (ppb)			
Median (range)	21.4 (8.4–38.1)	23.6 (10.6–28.1)	0.62
O_3_ (ppb)			
Median (range)	37.3 (8.7–62.9)	42.4 (29.3–68.3)	0.20
Daily mean surface pressure (hPa)			
Median (range)	1008.70 (998.38–1023.52)	1008.21 (1004.91–1020.21)	0.59
Daily average rainfall (mm)			
Median (range)	0.00 (0.00–105.90)	0.00 (0.00–3.60)	0.48
Daily average relative humidity (%)			
Median (range)	68.17 (44.09–88.28)	65.63 (59.82–72.19)	0.38
Daily maximum temperature (°C)			
Median (range)	28.55 (17.66–32.85)	29.94 (24.40–33.48)	0.40
Daily average temperature (°C)			
Median (range)	26.65 (16.06–30.93)	25.96 (19.76–30.31)	0.89
Daily minimum temperature (°C)			
Median (range)	23.89 (11.14–29.19)	21.55 (15.73–28.15)	0.78
Lag 3			
PM_2.5_ (μg/m^3^)			
Median (range)	36.9 (11.8–71.4)	37.2 (21.7–81.9)	0.74
NO_2_ (ppb)			
Median (range)	20.0 (9.1–35.2)	19.8 (13.3–31.1)	0.83
O_3_ (ppb)			
Median (range)	38.8 (12.8–61.1)	39.4 (30.6–68)	0.45
Daily mean surface pressure (hPa)			
Median (range)	1008.45 (997.68–1022.37)	1009 (1006.56–1019.61)	0.34
Daily average rainfall (mm)			
Median (range)	0.00 (0.00–518.02)	0.01 (0.00–5.46)	0.69
Daily average relative humidity (%)			
Median (range)	69.31 (40.57–83.82)	63.58 (57.15–74.64)	0.39
Daily maximum temperature (°C)			
Median (range)	28.55 (16.73–33.13)	28.63 (23.45–33.16)	0.81
Daily average temperature (°C)			
Median (range)	26.17 (14.90–30.61)	24.99 (19.17–30.68)	0.82
Daily minimum temperature (°C)			
Median (range)	23.79 (10.95–29.02)	20.98 (15.10–28.82)	0.63
Lag 4			
PM_2.5_ (μg/m^3^)			
Median (range)	37.2 (12.8–91.9)	36.7 (18.4–64.1)	0.69
NO_2_ (ppb)			
Median (range)	18.9 (6.2–35.9)	22.03 (8.6–35.6)	0.43
O_3_ (ppb)			
Median (range)	38.2 (4.4–59.2)	34.9 (12.7–73.4)	0.97
Daily mean surface pressure (hPa)			
Median (range)	1008.72 (994.40–1018.52)	1011.87 (1005.75–1018.86)	0.11
Daily average rainfall (mm)			
Median (range)	0.00 (0.00–133.32)	0.00 (0.00–49.37)	0.81
Daily average relative humidity (%)			
Median (range)	70.04 (49.80–83.26)	63.78 (54.15–71.03)	0.06
Daily maximum temperature (°C)			
Median (range)	29.14 (13.93–34.03)	26.17 (21.92–33.85)	0.59
Daily average temperature (°C)			
Median (range)	26.54 (13.05–31.10)	23.82 (18.29–31.07)	0.56
Daily minimum temperature (°C)			
Median (range)	24.00 (10.87–29.19)	21.24 (14.21–28.88)	0.48

Abbreviation: SPM, spontaneous pneumomediastinum.

**FIGURE 5:**
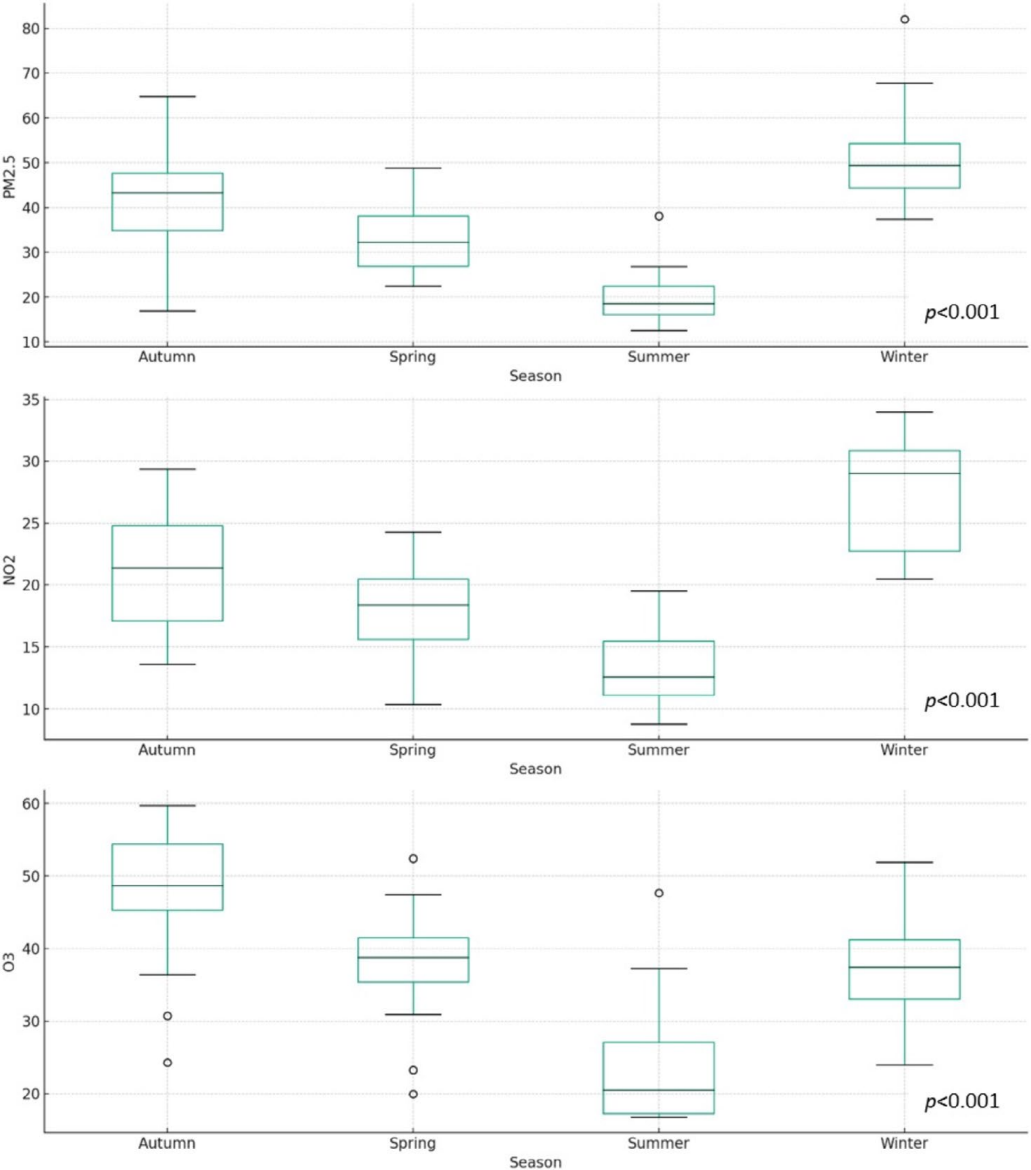
Seasonal distribution and comparison of air pollutant levels using box plot analysis. Box plots illustrate the distribution of PM_2.5_, NO_2_, and O_3_; concentrations across the four seasons (spring, summer, autumn, and winter). This analysis highlights seasonal variations in pollutant levels, with significantly higher concentrations observed during autumn and winter. PM2.5, fine particulate matter ≤ 2.5 μm; NO_2_, nitrogen dioxide; O_3_, ozone.

The seasonal distribution of air pollutants was also examined (see Figure 5). Notably, levels of PM_2.5_, NO_2_, and O_3_ were significantly higher in autumn and winter compared to spring and summer (*p* < 0.001). Consistent with these findings, Figure 3C,D, demonstrate that SPM incidence followed a seasonal pattern, with significantly greater occurrences in autumn and winter than in spring and summer (*p* < 0.001).

Table 5 summarizes the relationships between air pollutant levels and seasonal variation as predictors of SPM. Among all air pollutants, PM_2.5_ showed the strongest association with SPM onset in autumn and winter (entire case period: *β* = 1.15, 95% CI [0.27, 2.20], *p* = 0.012; and *β* = 1.18, 95% CI [0.31, 2.10], *p* = 0.010, respectively).

**TABLE 5:** Linear regression analysis of air pollutant levels as predictors of SPM with seasonal variation.

Factors	PM_2.5_ (μg/m^3^)		NO_2_ (ppb)		O_3_ (ppb)	
	*β* coefficient (95% CI)	*p*	*β* coefficient (95% CI)	*p*	*β* coefficient (95% CI)	*p*
Spring						
Case period (0–4 Day)	0.27 (−0.25–0.89)	0.30	0.23 (−0.39–0.86)	0.46	0.23 (−0.41–0.89)	0.46
Lag 0	0.36 (−0.10–0.82)	0.12	0.40 (−0.17–0.96)	0.16	0.33 (−0.30–0.95)	0.30
Lag 1	0.31 (−0.16–0.78)	0.19	0.20 (−0.37–0.76)	0.48	0.20 (−0.38–0.77)	0.48
Lag 2	0.29 (−0.23–0.81)	0.26	0.19 (−0.40–0.79)	0.52	0.24 (−0.34–0.82)	0.41
Lag 3	0.26 (−0.19–0.70)	0.24	0.23 (−0.36–0.81)	0.44	0.19 (−0.42–0.81)	0.53
Lag 4	0.31 (−0.22–0.83)	0.24	0.26 (−0.33–0.86)	0.38	0.46 (−0.16–1.10)	0.14
Summer						
Case period (0–4 Day)	0.46 (−0.25–1.18)	0.19	0.46 (−0.61–0.15)	0.38	0.49 (−0.07–1.07)	0.84
Lag 0	0.33 (−0.32–0.98)	0.30	0.48 (−0.43–1.39)	0.28	0.47 (−0.08–1.10)	0.08
Lag 1	0.58 (−0.12–1.28)	0.09	0.70 (0.18–1.23)	0.01	0.73 (0.18–1.23)	0.11
Lag 2	0.59 (−0.05–1.22)	0.07	0.59 (−0.33–1.51)	0.20	0.50 (−0.01–1.01)	0.05
Lag 3	0.45 (−0.17–1.08)	0.15	0.41 (−0.43–1.30)	0.32	0.41 (−0.11–0.94)	0.12
Lag 4	0.41 (−0.24–1.06)	0.21	0.56 (−0.36–0.15)	0.22	0.43 (−0.13–0.96)	0.11
Autumn						
Case period (0–4 Day)	1.15 (0.27–2.20)	0.012	0.94 (−0.09–1.98)	0.07	0.78 (−0.11–0.17)	0.08
Lag 0	0.91 (0.29–0.15)	0.006	0.91 (0.01–1.81)	0.04	0.69 (−0.01–1.39)	0.05
Lag 1	0.98 (0.28–1.69)	0.008	0.59 (−0.09–1.29)	0.09	0.59 (−0.09–1.29)	0.09
Lag 2	0.92 (0.15–1.69)	0.021	0.61 (−0.28–1.50)	0.17	0.79 (0.14–1.45)	0.02
Lag 3	1.16 (0.32–1.99)	0.009	1.10 (0.07–2.10)	0.37	0.66 (−0.15–1.48)	0.11
Lag 4	0.69 (−0.03–1.43)	0.06	0.76 (−0.02–1.53)	0.06	0.62 (−0.09–1.33)	0.09
Winter						
Case period (0–4 day)	1.18 (0.31–2.10)	0.010	1.30 (−0.01–2.63)	0.05	0.47 (−0.35–1.29)	0.25
Lag 0	1.04 (0.20–1.85)	0.015	0.65 (−0.53–1.80)	0.27	0.35 (−0.24–0.93)	0.24
Lag 1	1.01 (0.08–1.93)	0.034	0.05 (−0.64–0.75)	0.87	0.05 (−0.64–0.75)	0.87
Lag 2	1.34 (0.48–2.19)	0.003	1.27 (−0.05–2.60)	0.06	0.53 (−0.34–1.41)	0.22
Lag 3	0.78 (0.13–1.42)	0.02	1.46 (0.39–2.53)	0.11	0.61 (−0.17–1.40)	0.12
Lag 4	1.07 (0.37–1.76)	0.004	0.13 (0.19–0.23)	0.02	0.87 (0.28–0.15)	0.005

Abbreviation: SPM, spontaneous pneumomediastinum.

In addition, we conducted a comprehensive review of large case‐based studies (≥ 40 cases) published over the past two decades, as summarized in Table S1. The sex distribution, age profile, and triggering events observed in our study are largely consistent with findings from these prior studies. Notably, only one study has briefly mentioned seasonal variation in the context of spontaneous pneumomediastinum (SPM). However, although seasonal trends were observed, they were neither systematically analyzed nor emphasized as a primary focus of the study [2].

## 4 Discussion

In this 12‐year retrospective cohort study, we analyzed 70 cases of spontaneous pneumomediastinum (SPM) to assess the association between environmental factors, seasonal variation, and SPM occurrence. To our knowledge, this is the first study to investigate the impact of environmental factors on SPM incidence. Moreover, given the global variability in SPM presentation and the absence of established diagnostic and management guidelines, we focused on pneumorrhachis—a potentially severe and rare coexistence of SPM—in addition to revisiting commonly explored precipitating factors and diagnostic approaches.

In this study, a comparison between patients with and without pneumorrhachis revealed no significant differences in age, sex, smoking status, BMI, or the clinical assessments and treatments received. Although patients with pneumorrhachis had longer hospital stays, a higher incidence of severe‐grade SPM, and a greater likelihood of having identifiable trigger factors, their clinical course remained benign, with all making an uneventful recovery. Notably, pneumorrhachis can only be diagnosed when CT scans are performed in patients with SPM. In our study, all 70 included patients underwent CT scans, yielding a pneumorrhachis diagnosis rate of 12.8% (9/70), which is slightly higher than the previously reported incidence of 5.8% to 9.5% among SPM patients [13, 15]. Some studies emphasize the importance of differentiating primary SPM from secondary causes, such as esophageal perforation, through CT scans or esophagograms [1, 4, 6]. Conversely, other studies argue that extensive diagnostic workups—including bronchoscopy, esophagogram, esophagogastroduodenoscopy (EGD), additional CT scans, and antibiotic therapy—often do not significantly impact patient management and may lead to unnecessary interventions [3, 10, 11]. Our findings align with the existing literature. However, we believe that the use of CT scans holds significant value in accurately diagnosing both SPM and pneumorrhachis. Beyond diagnosis, CT imaging may offer insights into the severity of SPM and contribute to a more comprehensive understanding of its progression and overall clinical picture.

Another important issue is that a considerable number of SPM cases are linked to events that cause a sudden rise in intrathoracic pressure, including Valsalva maneuvers, intense physical activity, forceful coughing, vomiting, and severe asthma attacks [1, 2, 3, 4, 5, 6, 7, 8, 9, 10, 11]. However, many patients develop SPM without any identifiable triggering event [2, 4, 7, 8]. Air pollutants are known to irritate airway epithelial cells, leading to airway inflammation, dysfunction, and obstruction. Oxidative stress induced by NO_2_, O_3_, and PM_2.5_ plays a key role in driving inflammation in lung epithelial cells [23]. Previous studies suggest that these pollutants, along with meteorological factors, may impact lung parenchyma and contribute to spontaneous pneumothorax occurrence [24, 25]. Similarly, when communication with the lower airway is disrupted—either temporarily due to bronchospasm or airway inflammation—air may become trapped within the alveoli. In such cases, a sudden increase in the intrathoracic pressure gradient can rapidly distend weakened alveolar walls, ultimately leading to their rupture. The resulting air leakage then tracks along the bronchovascular sheaths into the mediastinum (Macklin effect), which might explain the pathophysiology of spontaneous pneumomediastinum (SPM) occurrence.

Our study identified significantly higher levels of PM_2.5_, NO_2_, and O_3_ during autumn and winter compared to spring and summer (*p* < 0.001), a pattern that is consistent with the well‐established seasonality of air quality in Taiwan. Tsai et al. previously reported that air pollution levels tend to be lowest in summer, gradually rising in colder months and peaking in winter, with central and southern Taiwan experiencing over 70 days of poor air quality annually [26]. In parallel, we observed a similar seasonal trend in the incidence of SPM, with significantly more cases occurring in autumn and winter (*p* < 0.001). When examining the relationship between seasonal variation in pollutant levels and SPM incidence, the standardized *β* coefficients for PM_2.5_ (*β* = 1.15 and *β* = 1.18, respectively) suggest a relatively stronger association during colder seasons. Although these findings are statistically significant, we acknowledge that they stem from observational data and should be interpreted as exploratory rather than causal. Prior epidemiological research has linked PM_2.5_ exposure to increased cardiopulmonary morbidity and mortality in Taiwan [27], as well as to asthma onset and exacerbations in both adults and children [28, 29]. In our study, asthma attacks were more frequently observed among pneumorrhachis patients compared to non‐pneumorrhachis patients (33% vs. 8.2%), suggesting a possible connection between air pollution, asthma exacerbation, and more severe SPM presentations. Nonetheless, we caution that this association requires further validation through prospective studies with larger sample sizes and more granular exposure assessment.

This study provides valuable data from southern Taiwan, a region with significant air pollution, contributing to the limited body of research on environmental factors and SPM occurrence. However, several limitations should be acknowledged. The retrospective design and relatively small sample size, particularly for pneumorrhachis cases, limit the statistical power of our analysis. Additionally, the relatively low incidence of pneumorrhachis in our study may have introduced selection bias. Despite these limitations, our findings lay the groundwork for future research on the relationship between SPM and environmental factors, particularly in patients with identifiable triggers who are exposed to higher regional air pollution levels (e.g., PM_2.5_). Moreover, our study did not include SPM cases from the COVID‐19 pandemic period (2020–2023), as this could have introduced selection bias, given that patients were less likely to seek medical care during this time. Furthermore, SPM has been reported in large cohorts of patients with severe pneumonia during the COVID‐19 pandemic [30, 31]. As the pathophysiology and clinical outcomes of COVID‐19‐associated SPM may differ significantly [32], careful consideration is needed when comparing cases from pandemic and non‐pandemic periods to avoid confounding effects.

In conclusion, spontaneous pneumomediastinum (SPM) appeared to exhibit seasonal clustering, with a higher incidence observed during autumn and winter. Although elevated PM_2.5_ levels were found to be more prominent in these seasons and showed a statistically significant association with SPM onset in our subgroup analysis, this relationship should be interpreted with caution. These findings suggest a possible connection between seasonal air pollution and SPM occurrence; however, further prospective studies are warranted to clarify this association and investigate potential underlying mechanisms. Additionally, while patients with pneumorrhachis presented with more identifiable triggers and required longer hospitalization, their clinical course remained benign and comparable to those without pneumorrhachis. Continued research is needed to improve understanding of the environmental and clinical contributors to SPM and to support more tailored management strategies.

## Ethics Statement

The study was conducted in accordance with the Declaration of Helsinki and was approved by the Ethics Committee of Kaohsiung Medical University Hospital (Approval no. KMUHIRB‐E(I)‐20180071).

## Conflicts of Interest

The authors declare no conflicts of interest.

## Supporting information


**Table S1:** Summary of large case‐based studies on spontaneous pneumomediastinum published between 2005 and 2025.

## Data Availability

The datasets used and/or analyzed during the current study are available from the corresponding author on reasonable request.
