# Supplementary material for: Impact of Environmental and Seasonal Factors on Spontaneous Pneumomediastinum With and Without Pneumorrhachis
Source: Kaohsiung J Med Sci. 2025 Aug 17;42(1):e70096. doi: 10.1002/kjm2.70096 (PMC12782255; doi:10.1002/kjm2.70096)
Supplement: Supplementary file 1 — Table S1: Summary of large case‐based studies on spontaneous pneumomediastinum published between 2005 and 2025. [file KJM2-42-e70096-s001.docx]

**Supplemental Table 1.** Summary of large case-based studies on spontaneous pneumomediastinum published between 2005 and 2025

| Author | Publication year | Study period | Number of patients | Sex (male) | Age (*) | Smoking | Triggering event | Asthma history | Seasonal variation | Country |
| --- | --- | --- | --- | --- | --- | --- | --- | --- | --- | --- |
| Potz et al. [1] | 2017 | 2004–2013 | 225 | 136 (60.4%) | 25 (17.6–57.6) | NA | NA | 31 (13.8%) | NA | USA |
| Kim et al. [2] | 2015 | 1996–2012 | 64 | 71 (80%) | 18.5 ± 4.0 | 7 (11%) | Coughing: 12 (18.8%)  Feeding: 9 (14.1%)  Vomiting: 7 (10.9%)  Exercise: 4 (6.3%)  Playing instruments: 1 (1.6%)  Unknown: 34 (53.1%) | 4 (6.3%) | Spring: 15 (23.4%)  Summer: 28 (43.8%)  Fall: 16 (25%)  Winter: 5 (7.8%) | South Korea |
| Yamairi et al. [3] | 2021 | 2005–2020 | 71 | 53 (75%) | 19.3 ± 6.4 | 7 (10%) | Exercise: 18 (25.3%)  Asthma attack: 13 (18.3%)  Shouting: 7 (9.8%)  Singing: 4 (5.6%)  Coughing: 3 (4.2%)  Vomiting: 2 (2.8%)  Playing instruments: 2 (2.8%)  Unknown: 22 (31%) | NA | NA | Japan |
| Song et al. [4] | 2017 | 2006–2015 | 45 | 35 (77.8%) | 18.9 ± 4.7 | 8 (17.8%) | Coughing: 4 (8.9%)  Exercise: 3 (6.7%)  Vomiting: 2 (4.4%)  Unknown: 36 (80%) | 1 (2.2%) | NA | South Korea |
| Yu et al. [5] | 2023 | 2010–2021 | 237 | 222 (93.7%) | 22 (21–25) | 92 (38.8%) | Coughing: 72 (30.4%)  URI: 66 (27.8%)  Physical exertion: 56 (23.6%)  Vomiting: 34 (14.3%) | 99 (41.7%) | NA | South Korea |
| Morgan et al. [6] | 2023 | 2001–2019 | 100 | 70 (70%) | 25.0 ± 8.5 | 11 (11%) | Coughing: 34 (34%)  Asthma attack: 27 (27%)  Retching: 24 (24%)  Strenuous activity: 11 (11%) | 27 (27%) | NA | USA |
| Macia et al. [7] | 2007 | 1990–2006 | 41 | 34 (83%) | 21.3 ± 4.8 | 14 (34%) | Exercise: 5 (12.2%)  Vomiting: 4 (9.8%)  Coughing: 3 (7.3%)  URI: 3 (7.3%)  Miscellaneous: 5 (12.2%)  Unknown: 21 (51.2%) | 9 (22%) | NA | Spain |
| Wong et al. [8] | 2013 | 2002–2011 | 87 | 59 (67.8%) | 12.3 ± 5.3 | NA | Idiopathic: 43 (49.4%)  URI: 12 (13.8%)  Asthma attack: 15 (17.2%)  Airway choking: 11 (12.6%)  Coughing: 3 (3.4%)  Croup: 2 (2.3%)  Physical exertion: 1 (1.1%) | NA | NA | Taiwan |
| Perna et al. [9] | 2010 | 2000–2008 | 47 | 33 (70.5%) | 27.3 | 13 (27.6%) | Drug inhalation: 26 (55.3%)  Exercise: 7 (14.9%)  URI: 6 (12.7%)  Coughing: 4 (8.5%)  Vomiting: 3 (6.4%)  Unknown: 1 (2.1%) | 8 (17%) | NA | Spain |
| Bakhos et al. [10] | 2014 | 2006–2011 | 49 | 26 (53%) | 19 ± 9 | 11 (22%) | Coughing: 14 (29%)  Vomiting: 8 (16%)  Physical exertion: 3 (6%)  Drugs: 3 (6%)  Panic attack: 1 (2%)  Unknown: 20 (41%) | 20 (41%) | NA | USA |
| Wald et al. [11] | 2024 | 2011–2021 | 166 | 118 (72.8%) | 18 (15–21) | NA | Coughing: 64 (38.5%)  Vomiting: 64 (38.5%)  Asthma attack: 16 (9.6%) | 64 (38.5%) | NA | USA |
| Liu et al.  (this study) | 2025 | 2007–2019 | 70 | 57 (81.4%) | 21.1 ± 11.4 | 10 (14.3%) | URI: 9 (12.9%)  Asthma attack:8 (11.4%)  Coughing: 5 (7.1%)  Exercise: 5 (7.1%)  Vomiting: 4 (5.7%)  Playing instruments: 1 (1.4%)  Unknown: 38 (54.3%) | NA | Spring: 13 (18.6%)  Summer: 10 (14.3%)  Fall: 22 (31.4%)  Winter: 25 (35.7%) | Taiwan |

(*): mean *±* SD or median with interquartile range; NA: not available; URI: upper respiratory tract infection
